# High Diversity and Low Specificity of Chaetothyrialean Fungi in Carton Galleries in a Neotropical Ant–Plant Association

**DOI:** 10.1371/journal.pone.0112756

**Published:** 2014-11-14

**Authors:** Maximilian Nepel, Hermann Voglmayr, Jürg Schönenberger, Veronika E. Mayer

**Affiliations:** 1 Division of Structural and Functional Botany, Department of Botany and Biodiversity Research, University of Vienna, Vienna, Austria; 2 Division of Systematic and Evolutionary Botany, Department of Botany and Biodiversity Research, University of Vienna, Vienna, Austria; 3 Institute of Forest Entomology, Forest Pathology and Forest Protection, Department of Forest and Soil Sciences, BOKU-University of Natural Resources and Life Sciences, Vienna, Austria; Georg-August-University Göttingen, Germany

## Abstract

New associations have recently been discovered between arboreal ants that live on myrmecophytic plants, and different groups of fungi. Most of the – usually undescribed – fungi cultured by the ants belong to the order Chaetothyriales (Ascomycetes). Chaetothyriales occur in the nesting spaces provided by the host plant, and form a major part of the cardboard-like material produced by the ants for constructing nests and runway galleries. Until now, the fungi have been considered specific to each ant species. We focus on the three-way association between the plant *Tetrathylacium macrophyllum* (Salicaceae), the ant *Azteca brevis* (Formicidae: Dolichoderinae) and various chaetothyrialean fungi. *Azteca brevis* builds extensive runway galleries along branches of *T. macrophyllum*. The carton of the gallery walls consists of masticated plant material densely pervaded by chaetothyrialean hyphae. In order to characterise the specificity of the ant–fungus association, fungi from the runway galleries of 19 ant colonies were grown as pure cultures and analyzed using partial SSU, complete ITS, 5.8S and partial LSU rDNA sequences. This gave 128 different fungal genotypes, 78% of which were clustered into three monophyletic groups. The most common fungus (either genotype or approximate species-level OTU) was found in the runway galleries of 63% of the investigated ant colonies. This indicates that there can be a dominant fungus but, in general, a wider guild of chaetothyrialean fungi share the same ant mutualist in *Azteca brevis*.

## Introduction

It is now clear that microorganisms are major partners in obligate interactions between ants and plants. Ant–fungus associations have been recognised since the mid-19th century (e.g. [1]), and the best-studied examples are the fungal gardens of the leaf-cutter ants in the tribe Attini. Leaf-cutter ants grow monocultures of basidiomycetes on shredded leaf material and feed on the nutrient-rich tips of the fungal hyphae [2], [3]. Other examples of ant–plant–fungus interactions have also been found recently in different groups of non-attine ants, where ascomycete fungi are cultivated in domatia (nesting spaces provided by host plants) or on a cardboard-like construction material (named “carton” in ant-plant literature) [4]–[7]. Such ant–plant–fungus associations have been described from Africa, America and Asia and involve a wide range of plant lineages associated with an equally wide range of ant groups [7].

**Figure 1:**
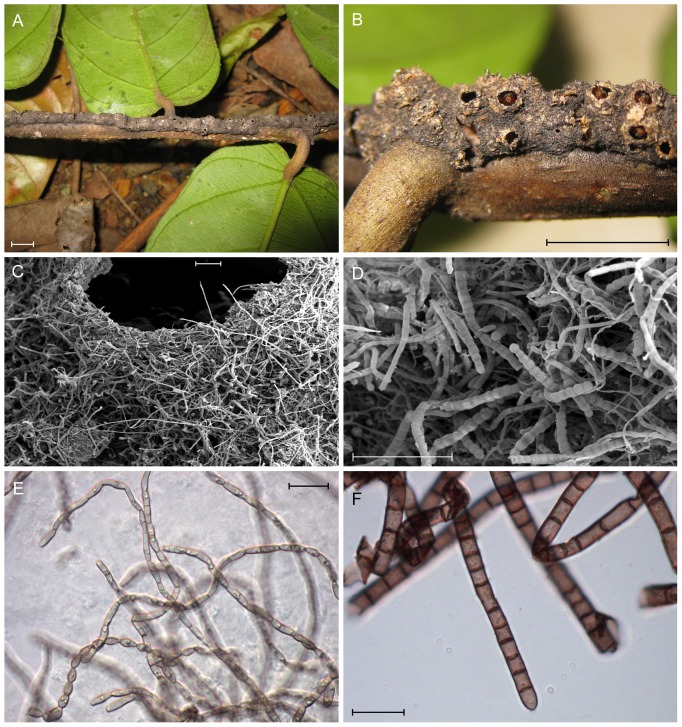
Carton runway galleries built by *Azteca brevis* ants, consisting of mycelia and various organic particles. (A, B) Galleries on the lower side of branches of *Tetrathylacium macrophyllum*: note the scattered circular openings in the gallery walls. (B) Alarmed workers wait with open mandibles below the holes for prey or intruders. (C, D) Scanning electron microscope images of the gallery walls infused with different types of hyphae. (E, F) Light-microscope images of both hyphal types: (E) thin-walled hyaline hyphae typical for carton clades 2 and 3; (F) pigmented thick-walled hyphae typical for carton clade 1 (see Figure 2–3). Bars: (A, B) 1 cm; (C, D) 100 µm; (E, F) 20 µm.

There is evidence that the fungi cultivated within the domatia are used as a food source [8], whereas those in the carton-like material do not appear to be consumed. Rather, they seem to serve to stabilise the carton mechanically. Carton structures with fungi were first documented in nest walls of the European ant *Lasius fuliginosus* inside hollow tree-trunks [1], [9], [10]. They have since been found in the walls of free-hanging canopy ant nests in the Palaeotropics [11], [12] and in the Neotropics, where ants use fungus-infused, carton-like material to construct tunnel systems called “runway galleries” along branches of their host trees [6], [13], [14] (Figure 1A–D).

In the tripartite ant–plant–fungus interactions involving non-attine ants studied so far, the vast majority of the fungi have belonged to the ascomycete order Chaetothyriales, the so-called “black yeasts” [7]. These are usually dark, melanised, slow-growing fungi that often colonise extreme environments [15]–[18], but little is known about the order's ecology and diversity.

A recent survey based on molecular phylogenetics showed that ant-associated chaetothyrialean fungi belong to four clades within the order: a domatia-symbiont clade, two clades with carton fungi, and a mixed clade containing both domatia symbionts and carton fungi [7]. Only a few isolates were placed outside these four clades, and ant-fungi cultivated in the domatia seemed to be specific to each ant species [7]. Carton structures have been less well investigated, and studies to date have produced disparate results: in the *Hirtella* (Chrysobalanaceae)/*Allomerus* (Formicidae) association, it was reported that a specific fungus is cultivated by the ants in the wall material of their galleries [14]; in contrast, a wider guild of fungi seems to be involved in the structurally analogous galleries of the *Tetrathylacium*/*Azteca* association [6]. The aim of the present investigation was (1) to unravel the diversity and geographical pattern of the carton fungi found in the carton galleries, and (2) to investigate the hyphal morphology of the relevant fungal strains with respect to the proposed function of the galleries.

## Material and Methods

### Species and study site

*Azteca brevis* Forel, 1899 (Formicidae, Dolichoderinae) is a reddish-brown ant, c. 4 mm long, known from wet forests of the southern Pacific lowlands of Costa Rica [19]. Colonies have been found on *Tetrathylacium macrophyllum* (Salicaceae), *Licania* sp. (Chrysobalanaceae), *Grias* sp. (Lecythidaceae), *Myriocarpa* sp. (Urticaceae), *Ocotea nicaraguensis* (Lauraceae) [19] and *Lonchocarpus* sp. (Fabaceae). The nesting chambers inside the stems are connected externally by runway galleries dotted with small, circular holes (Figure 1A, B).

The most common host plant for *Azteca brevis* is *Tetrathylacium macrophyllum* Poepp. (Salicaceae), a small tree (c. 8 m) that grows on the Pacific slopes of Central and South America in areas characterised by high annual rainfall (>5000 mm). It is found chiefly on steep slopes near rivers and streams in primary forest [20]. About 30% of *T. macrophyllum* trees are occupied by *Azteca brevis* [21], [22]. The ants start by colonising hollow chambers in the branches that the plant forms through pith degeneration. As the colony grows, the ants excavate the remaining pith between adjacent naturally formed chambers, and build large nest sites inside the branches.

We sampled within a 5-km circle around the Tropical Research Station La Gamba, Costa Rica (8° 42′ 03″ N, 83° 12′ 06″ W) along the Waterfall Trail, Bird Trail, Río Gamba, Río Bolsa and Río Sardinal. Carton samples, ants and plant parts for herbarium specimens were collected from 18 *T. macrophyllum* trees and one *Lonchocarpus* tree colonised by *Azteca brevis*, under permission from SINAC – Sistema Nacional de Areas de Conservación de Costa Rica of the Ministry of Environment and Energy (MINAE) to M.N. and V.E.M. (No. 182-2010-SINAC). In recent years, trees colonised by *Azteca brevis* have become inexplicably rare at the study site (VEM, pers. obs.), limiting the sample size to 19 trees colonised by *Azteca brevis*. At least three carton pieces per tree and colony, each c. 1 cm long, were taken from runway galleries and stored in 1.5-mL reaction tubes sealed with air-permeable cotton wool. The reaction tubes were kept in a sealed plastic bag with silica gel for three weeks before culturing the fungi; the bags were transported and stored at room temperature.

### Fungal cultures and DNA-extraction

At the University of Vienna, a c. 10-mm^2^ piece of carton was placed into a droplet (c. 20 µL) of sterile water and fragmented with sterile forceps to make a mycelial suspension. An aliquot of mycelial suspension was then diluted with 1 mL sterile water and spread over each of two 2% malt extract agar plates (MEA) containing 0.5% penicillin and 0.5% streptomycin. This was carried out on average for three samples per colonised tree. The plates were stored at room temperature and visually checked under a dissecting microscope at least once a day for 11 days. Fast-growing “weeds” (*Aspergillus*, *Cladosporium*, *Fusarium*) were excised to prevent overgrowth of the slower-growing carton fungi. The thick, darkly pigmented hyphae that are typical for the carton usually started to grow after 2–4 days and were then transferred to new 2% malt extract agar plates. Several carton samples from each ant colony and tree were processed in this way to minimise any cultivation bias.

Sections of approximately 25 mm^2^ were cut out from mycelia on pure-culture agar plates and stored in 2-mL reaction tubes at −20°C. The frozen samples were subsequently freeze-dried overnight and ground with five glass beads (3 mm diameter) for 10 min at 30 Hz in an MM 400 mixer mill (Retsch, Germany), after which DNA was extracted using NucleoSpin 96 Plant II kits (Macherey-Nagel, Düren, Germany).

### PCR and cleanup

A 1.5–3.5-kb nuclear ribosomal DNA (rDNA) fragment comprising partial small subunit (SSU), complete ITS1–5.8S–ITS2 (ITS) and partial long subunit (LSU) sequences was amplified with the fungal primers V9G [23] and LR5 [24] using Thermo Scientific 2.0× ReddyMix Extensor PCR Master Mix and 1.1× ReddyMix PCR Master Mix (ABgene, Epsom, UK) (for primer sequences and detailed PCR protocol, see Table S1 in Appendix S1). The PCR products were purified with 6 U exonuclease I and 0.6 U FastAP thermosensitive alkaline phosphatase (Fermentas, St. Leon-Rot, Germany) [25]; the PCR product was then incubated for 30 min at 37°C, followed by enzyme deactivation for 15 min at 85°C.

### Sequencing

DNA was cycle-sequenced with ABI PRISM BigDye Terminator Cycle Sequencing Ready Reaction v. 3.1 (Applied Biosystems, Warrington, UK) using the PCR primers and primers LR3 [24] and ITS4 [26]. For sequences with large indels, the additional primers LR2R-A, LR2-A [27], F5.8Sr, F5.8Sf [28] and LR3-CH (5′-GGT ATA GGG GCG AAA GAC TAA TC-3′) were necessary to obtain full-length sequences (see Appendix S1 for detailed sequencing protocol). Sequencing was performed on an ABI 3730xl Genetic Analyzer automated DNA sequencer (Applied Biosystems). One sequence of each genotype was deposited in GenBank. The complete list of accession numbers for the SSU–ITS–LSU locus can be found in Table S2 in Appendix S1.

### Analysis of sequence data

After a BLAST search (Basic Local Alignment Search Tool in GenBank, http://blast.ncbi.nlm.nih.gov/Blast.cgi) of the nuITS1–5.8S–ITS2–LSUr DNA sequences obtained from the ant carton (423 in total), 381 sequences were identified as Chaetothyriales and used for further analyses. For phylogenetic analyses, identical sequences from carton samples were reduced to a single sequence per genotype. Cases where two sequences differed only in homopolymer regions were also merged to a single genotype.

For alignment, the chaetothyrialean sequences used in Voglmayr *et al.* [7] and the closest sequences to our isolates from GenBank were added. *Verrucaria denudata*, *V. csernaensis* and *V. andesiatica* (Verrucariales) were included as outgroups. Ambiguously aligned regions in ITS1/ITS2 and leading gap regions were excluded. The matrix of 258 sequences contained 7767 alignment positions, with the longest sequence comprising 3425 nucleotides. Alignments were produced with Muscle 3.8.31 [29] and revised in BioEdit 7.1.3.0 [30]. (GenBank accession numbers of the sequences included in the phylogenetic analyses are listed in Table S2 in Appendix S1.)

For Bayesian analyses, MrBayes 3.2.1 [31] was run through the Bioportal web service of the University of Oslo [32]. The six-parameter general time-reversible substitution model was used, with a proportion of invariant sites and a gamma distribution for the remaining sites (GTR + I + G), as determined by Modeltest 3.7 [33]. Three parallel runs of four chains were performed over 30 million generations, sampling 30 000 trees in each run. For 90% majority-rule consensus trees, the first 2000 trees of each run were discarded as burn-in.

Maximum-parsimony (MP) bootstrap analyses were performed with PAUP* 4.0b10 [34], using 1000 replicates of a heuristic search with 10 rounds of random sequence addition during each bootstrap replicate and a limit of 100 000 rearrangements per replicate. TBR branch swapping was used, allowing multitrees, and steepest descent was set to ‘no’. Gaps were treated as missing data, and no weighting of nucleotides was applied.

The maximum-likelihood (ML) analyses used RAxML [35], as implemented in the programme raxmlGUI 0.95 [36]. The GTRGAMMAI nucleotide substitution model was applied for the ML heuristic search and the ML rapid bootstrap analysis.

### Fungal distribution at the genotype and species levels

To evaluate the specificity of the fungi to *Azteca brevis*, the frequency of occurrence on the sampled trees was analyzed. For each tree, a matrix containing genotypes and sampled trees was compiled (Table S3 in Appendix S1) and Bray–Curtis similarity indices among sampled trees were plotted by non-metric multidimensional scaling (NMDS) with 1000 restarts. In addition, an ANOVA of similarity (ANOSIM) was conducted with up to 999 permutations, based on different geographical study sites (Table S2 in Appendix S1). All analyses were carried out with Primer 5 5.2.9 (PRIMER-E, 2002).

Slightly different genotypes can, however, represent the same species: the maximum number of mutations between two individuals of the same species (mutation limit) varies with the DNA region and type of organism. For fungi, the ITS region is more variable than SSU or LSU [37]. Because ITS is the main DNA fragment in the present study, the mutation limit was determined by multiplying the maximum intraspecific variation of ITS (0.58%) [37] by the average sequence length. We define an OTU as a species with a maximum genotype variation of 12.02 mutations. The abundance of OTUs was also analyzed using a modified presence–absence matrix (Table S4 in Appendix S1).

### Light microscopy and SEM analysis of carton material and fungal hyphae

Pieces of carton from 16 trees and aerial hyphae of the pure cultures were investigated using a Zeiss AxioImager A1 compound microscope with a Zeiss AxioCam ICc3 digital camera. SEM investigations were made with a Jeol JSM-T 300 scanning electron microscope (SEM) at 10 kV.

## Results

### Cultures

Pure cultures of carton fungi were obtained from carton material of host trees colonised by *Azteca brevis*. Because different species could not be distinguished morphologically, all the pigmented hyphae that germinated were transferred to MEA plates to obtain pure cultures. In total, 423 pure cultures were sequenced, resulting in 128 different genotypes after identical sequences were removed.

### Molecular phylogenetic analyses

**Figure 2:**
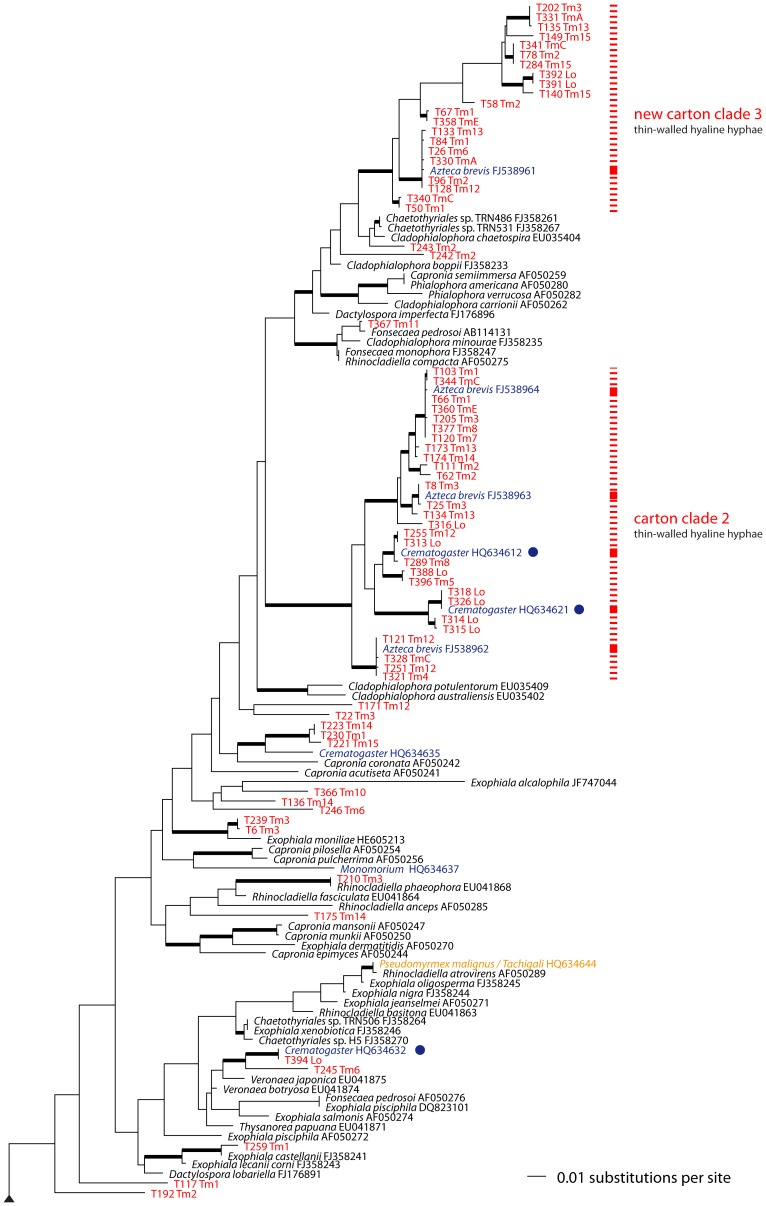
Phylogram of Chaetothyriales, top part. The maximum-likelihood tree is shown, based on partial SSU, complete ITS and 5.8S, and partial LSU rDNA regions. Bold branches are supported in all three analyses: BA probabilities higher than 0.9, ML and MP bootstrap support above 70%. Red labels denote fungal genotypes isolated in this study from ant-built carton structures on *Tetrathylacium macrophyllum* and *Lonchocarpus* sp. trees; orange and blue mark domatia fungi and carton fungi, respectively, from Voglmayr *et al.* [7]; GenBank accession numbers follow taxon names; solid red, violet and orange vertical lines indicate clade definitions and captions from Voglmayr *et al.* [7]; dotted lines mark clade extensions from this study. Blue dots point out three sequences from other continents (2× Cameroon; 1× Thailand) differing by only three mutations from our Costa Rican genotypes. Note the high diversity of isolated genotypes (the large clade extensions compared to Voglmayr *et al*. [7] are due to a greater number of samples and the new monophyletic carton-fungi cluster (new carton clade 3).The tree is continued in Figure 3.

**Figure 3:**
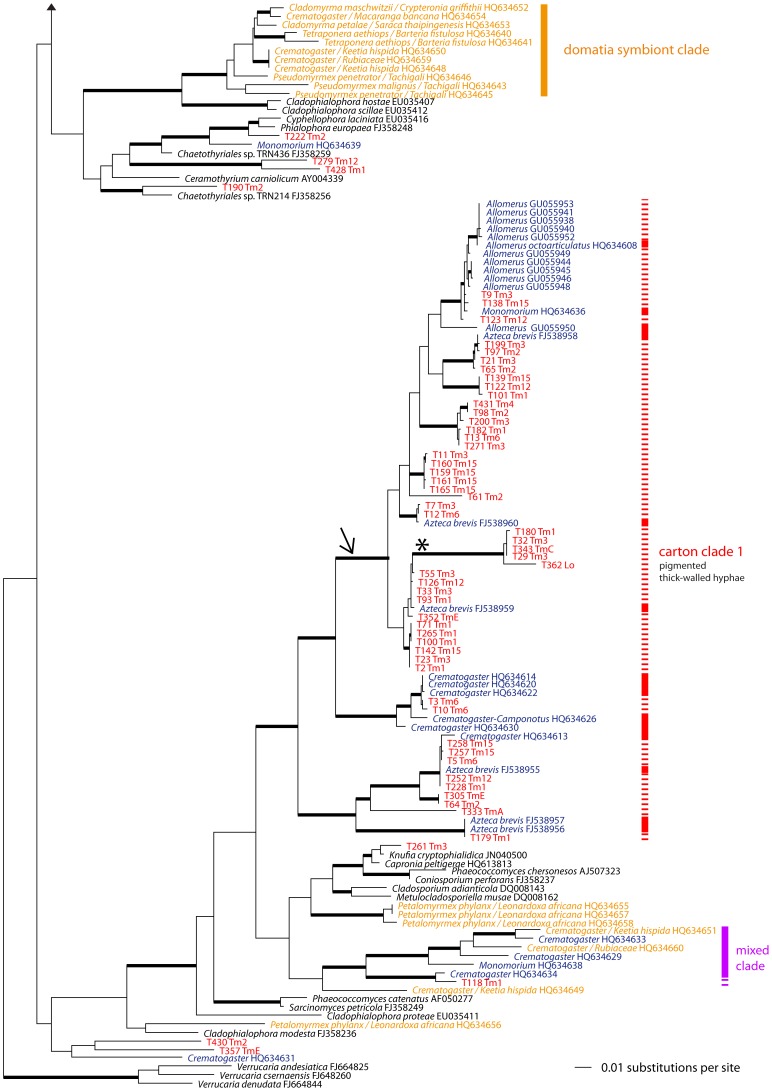
Phylogram of Chaetothyriales, bottom part. Continuation of Figure 2 with arrowheads indicating the connection. For label and colour descriptions see legend to Figure 2. The clade labelled with an asterisk (*) is placed more basally (arrow) in the MP analysis than in ML and BA analyses. Note the domatia-symbiont clade, which remains distinct from carton fungi.

After adding relevant sequences from GenBank, the final alignment, including outgroups, consisted of 258 sequences and 7767 alignment positions. In the best tree from the ML search (shown as a phylogram in Figure 2–3), backbone support is mostly low or absent, but most of the subclades are well-supported. Consensus trees across the three analyses differed topologically only in one point: one clade of five sequences containing a large indel of c. 1000 bp within the LSU region is located more basally in the topology resulting from the MP analysis than in the topologies from BA and ML analyses (marked with an asterisk and an arrow in Figure 3). Bootstrap support for this node in the MP analysis was only 73%, so this difference was not considered further.

The phylogenetic reconstruction revealed three main clades containing 73% (100 out of 128) of the isolated genotypes (Figure 2–3). Carton clades 1 and 2 were described by Voglmayr *et al.* [7], but carton clade 3, which was represented by a single sequence in previous analyses, is new. The remaining 28 genotypes are distributed across the phylogenetic tree of Chaetothyriales, except for the “domatia-symbiont clade”. Three sequences from carton material from Cameroon and Thailand are nearly identical to some fungi sequenced in this study. Each of those three fungal genotypes (marked by blue dots in Figure 2) differs by only three mutations from fungi grown from carton structures collected in Costa Rica.

### Fungal distribution at the genotype and species levels

The 128 different genotypes isolated from carton material of 19 *Azteca*-inhabited trees were analyzed with a presence–absence matrix (Table S3 in Appendix S1). The matrix showed that no genotype was found on all trees, with the most common one isolated from nine out of 19 trees. On average, 10.5 different genotypes occurred on each tree, of which 46% were unique to single trees. In the carton sample of one *Lonchocarpus* tree inhabited by *Azteca brevis*, 11 out of 12 genotypes (92%) were unique.

**Figure 4:**
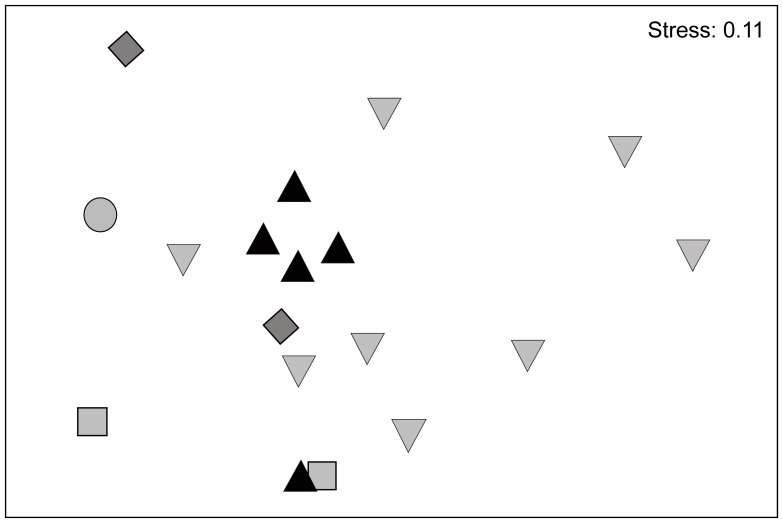
Correlation analysis of genotype sets and collection sites. Non-metric multidimensional scaling (NMDS) plot based on Bray–Curtis similarities between fungal genotype sets occurring on carton material of 18 sampled *Tetrathylacium macrophyllum* trees, and one *Lonchocarpus* sp., colonised by *Azteca brevis*. Different symbols represent trees from different collection sites (squares: Waterfall Trail; triangles: Bird Trail; circle: Río Gamba; diamonds: Río Bolsa; inverted triangle: Río Sardinal).

Bray–Curtis similarity indices between sampled trees were calculated to investigate the correlation between genotype composition and collection site. The non-metric multidimensional scaling (NMDS) plot showed no clustering of trees from the same collection site (Figure 4), and the analysis of similarity (ANOSIM) showed a significance level of only *P* = 0.32. A correlation between genotype composition and collection site can therefore be ruled out. One sampled tree (Tm10) had to be excluded because only a single, unique fungus could be isolated, and the Bray–Curtis distance to the other sampled trees was too great for Tm10 to be displayed without clustering all remaining trees too tightly together.

The results were similar at the approximated species level. The 128 genotypes were reduced to 62 OTUs, and the most common OTU in the modified presence–absence matrix, represented by nine genotypes, was found on 12 out of 19 trees (63%) (Table S4; Figure S1 in Appendix S1). Three other OTUs were found on a total of 9 out of 19 trees (47%). The correlation of fungal community and collection site at the species level is weak (NMDS plot, Figure S2 in Appendix S1) and not significant (ANOSIM: *P* = 0.16).

### Light microscopy of carton material

Dark, melanised moniliform hyphae with thick cell walls (Figure 1F) appeared to be dominant in each sample. The cell width of these thick-walled hyphae ranged from about 6 to 9 µm. Hyaline hyphae with a cell width less than 5 µm were also present, but appeared less abundant (Figure 1E). Aerial hyphae of pure cultures representing the three carton clades were also examined and, surprisingly, darkly pigmented, thick-walled hyphae are largely restricted to carton clade 1, whereas thin-walled hyaline hyphae are found in carton clades 2 and 3 (Figure 2–3).

## Discussion

There is growing evidence that multicellular organisms are shaped by symbioses with smaller partners – often microbial – that contribute to their host's nutrition, protection and even to their normal development [38]. In obligate interactions between ants and plants, for example, it has only recently become apparent that micro-organisms are major partners in interactions that go far beyond the relationship between the ant and the plant. In ant–plant symbioses from Africa, America and Asia, ascomycete fungi are cultivated in domatia and on ant-built carton structures, involving a wide range of distantly related ants and plant families [5], [7]. This study is, however, the first dealing with fungi in ant-built cardboard-like carton material in which pure cultures of several samples were made per colony. This resulted in 381 pure cultures and 128 chaetothyrialean genotypes, the highest number of carton-associated chaetothyrialean symbionts ever found associated with a single ant species.

This high number of fungus genotypes of the ascomycete order Chaetothyriales (“black yeasts”) is astonishing, because these fungi generally seem to have weak competitive abilities. They are slow-growing and often extremophilic. They occur on nutrient-poor substrates, such as leaf or rock surfaces [15], [39], or in toxic environments [17], [18], and quickly disappear under less extreme conditions [7]. It is not yet known why “black yeasts” are so dominant on the carton material of ants. The frequent germination of fast-growing “weeds” on isolation plates indicates that the spores of moulds probably occur on the carton surface, but that their growth is inhibited. It may be the gallery substrate, the weeding and grooming behaviour of *Azteca brevis*, some ant-specific compounds or antifungal substances released from the fungi themselves that cause this inhibition. One indication that the construction material also shapes the fungal community on the galleries is the fact that 11 of 12 genotypes (92%) isolated from the samples collected from carton on a *Lonchocarpus* tree were unique and not present on the *Tetrathylacium* trees. *Azteca brevis* workers use particles of bark, excavated pith tissue and epiphylls from the host trees as materials for gallery construction, and plant secondary compounds may disfavour fungi other than Chaetothyriales. Furthermore, *Azteca brevis* was observed to groom the carton galleries constantly, and even to nourish them (M. Nepel & V. Mayer, unpubl.). Antibacterial and antifungal compounds produced by ants' exocrine glands, such as the metapleural gland, may play an important role in preventing other moulds from growing [40], [41]. In contrast, Chaetothyriales are able to tolerate and even to metabolise aromatic hydrocarbons [18] and can therefore cope and may even use ant-produced antifungal compounds metabolically. Finally, Chaetothyriales themselves might produce bioactive substances against competing fungi [42]. The combination of these factors may account for the relationship between ants and those fungi.

### Molecular phylogenetic analyses and fungal diversity

There was no ubiquitous fungus (genotype or OTU) among the 128 genotypes found in this study; 78% of the sequenced carton fungi clustered into three clades. Two of those clades were already established [7], and we have discovered a third (Figure 2). The 28 genotypes that were not assigned to any of those clades were scattered across the whole phylogenetic tree (Figure 2–3), but none of the 128 fungal genotypes isolated from the carton samples arose in the “domatia-symbiont clade” [7]. The fungi belonging to this special clade are highly distinct from carton fungi in terms of their hyphal morphology and growth form (hyaline or less pigmented and tending to produce spores in domatia fungi) [7] probably due to their different functions. Domatia fungi are used as food for the larvae [8], whereas carton fungi probably improve the stability of carton walls in ant nests or runway galleries. The specificity and coevolutionary dynamics between domatia and carton symbionts may differ.

### Fungus specificity at the genotype and species level

In most known insect–fungus symbioses (e.g. termites [43], [44] or leaf-cutter ants [45]), the associated fungus is cultivated for food, whereas *Azteca brevis* is not likely to eat the fungi, but cultivates them for their nest architecture. In this association, the ants as a single host use a group of multiple fungus species for this purpose. The interpretation of the degree of specificity depends on the taxonomic level and differs between the fungi involved. At higher taxonomic levels, the interaction specificity between *Azteca brevis* and Chaetothyriales is high: chaetothyrialean fungi were found in every carton sample analyzed. At the genotype level, a much more modest degree of interaction specificity was seen (Table S3 in Appendix S1): the most common genotype (T121_Tm12) was associated with 9 out of 19 ant colonies (c. 47%). No correlation between fungal community and collection site was found, and the fungal community seems not to be habitat-specific (Figure 4). Merging genotypes into OTUs based on the maximum intraspecific variation of ITS [37] increased the occurrence of the most common fungus (represented by T66 in Table S4 in Appendix S1) to 63% of the ant colonies and the three next most common OTUs to 47% (Table S4 in Appendix S1). Surprisingly, a mean of eight different OTUs were found per carton sample, but no specialist fungal partner obligate to all *Azteca brevis/Tetrathylacium macrophyllum* associations was found. Environmental samples for the most common OTU could not be analyzed, because no primers could be developed which were specific enough for reliable separation of the OTUs, and only culturing was possible. It might be argued that culturing introduces a bias, in that a genotype could have been missed. Because we sampled the carton galleries of every colony and tree several times each, and also performed the isolation procedure on average three times per sample, it is unlikely that we missed any genotype. A heterogeneous spatial distribution of the genotypes along the branches can also be excluded, because we always took samples at positions from the branching point to the tip of a branch, and would therefore have included any heterogeneity.

Moreover, some fungal sequences from *Azteca brevis* carton are nearly identical at the SSU–ITS–LSU region with those of fungi from cartons of other ant species and from samples collected on other continents. Two sequences from *Crematogaster* carton nests from Cameroon (*Crematogaster* HQ634612 and *Crematogaster* HQ634621), and one found in Thailand (*Crematogaster* HQ634632) [7] differ by only three mutations from the Costa Rican sequences (blue dots in Figure 2). This indicates that at least some ant-associated chaetothyrialean fungi have a transcontinental distribution and are associated with more than one ant genus rather than being specific to *Azteca brevis* hosts.

### Fungus selection and origin of the fungi

The carton runway galleries of *Azteca brevis* are used as a defence against intruders and as ambush traps for capturing prey (V. Mayer, pers. observ.), analogous to the carton galleries described for *Allomerus decemarticulatus* [13]. The fungal symbionts may play a particularly important functional role by increasing the stability of the carton produced by the ants [6], [13]. The hypothesis that fungi are used for reinforcement was first raised by Lagerheim for the carton constructions of *Lasius fuliginosus* [10] and it has been accepted by many authors [6], [11], [13], [46]. Trimming and grooming of the carton fungi has been observed and, due to the reinforcement demand, it may be expected that *Azteca brevis* would select fungal symbionts with a particular phenotype. Because we conducted a pure-culture method, we were able to examine the hyphae microscopically and correlate genotype or OTU with hyphal type. Two types of hyphae were regularly found: thin-walled, hyaline hyphae (Figure 1E), which represent two of our three major carton clades. The other type, thick-walled, melanised hyphae (Figure 1F), are less abundant in the species in our phylogenetic tree. When investigating pieces of carton, however, the thick-walled hyphae visibly dominated the biomass. This indicates that the second hyphae type is favoured on the carton. This may be either due to the substrate or due to the ants' preference. Fungi with thick-walled, melanised hyphae are likely to be better for the carton's stability than thin-walled hyphae.

Unfortunately, no alate queens were found, and interpretations of the transmission of the fungi must therefore be made without experimental evidence. The lack of any strong specificity indicates that the fungi growing on the *Azteca brevis* carton are either transmitted horizontally or environmentally acquired, although the degree of host–symbiont specificity is not always correlated with the transmission mode [47]. *De novo* acquisition of fungal symbionts from the environment in each ant generation would, however, explain the high number of genotypes and OTUs in the carton samples. *Azteca brevis* workers may collect spores or hyphal fragments from the environment, as termites do [48], but detailed field observations of the worker ants' behaviour are needed to prove this. Also, the plant material used for construction (typically bark and epiphylls) may already be infected with chaetothyrialean spores. The branches and stems of trees inhabited by *Azteca brevis* appear to be completely cleaned of epiphylls (algae, bryophytes and lichens) and epiphytes. In fact, the newly described Chaetothyriales family Trichomeriaceae was found to grow on the surface of living leaves [49]. Investigation of the host plants' surface (stem, branches and leaves) and the epiphylls are needed to clarify whether Chaetothyriales are already present. The chaetothyrialean fungus community of the carton galleries might be a subset of the fungal community found on the host plant's surface.

### Two analogous systems: *Allomerus* sp. and *Azteca brevis*

A plant–ant–fungus association with runway galleries that are structurally similar to those in the present study is seen in Amazonian *Allomerus* ants living on *Hirtella physophora* (Chrysobalanaceae) and *Cordia nodosa* (Boraginaceae) [14]. In contrast to the *Azteca brevis* carton, with its guild of numerous species of Chaetothyriales, the *Allomerus* ants are described as cultivating one specific fungal symbiont on the carton galleries (see upper part of carton clade 1; Figure 3). *Allomerus* foundress queens apparently store a pellet with the specific carton fungus represented by a monophyletic group of haplotypes on the domatium wall. While building their runway galleries, *Allomerus* workers were observed to glue pellets from scraped epidermis and mesophyll of the inner domatia walls onto the gallery frame built from trichomes of the host plant [14]. In *Azteca brevis* colonies, no inoculation pellet was found, an observation that supports the hypothesis that fungal symbionts are acquired *de novo* from the environment.

Although the sampling methods were different (only newly produced carton material was collected from the *Allomerus* galleries, whereas it was mainly “mature” black material that was sampled from the *Azteca brevis* galleries), the frequency of the most common fungus or OTU was more or less the same: on 57% (138 out of 240) of the *Allomerus* galleries, and on 63% (12 out of 19) of the *Azteca brevis* galleries. A fungal species found in a little over half of the analyzed carton samples should not, however, be regarded as specific.

## Conclusions

We give an insight into the diversity of Chaetothyriales present in the carton galleries of *Azteca brevis*. Our results refute the initial hypothesis that *Azteca brevis* forms a symbiosis with a specific fungus. At the genotype level as well as that of approximated species (OTUs), the fungi we isolated appear to be a guild of different Chaetothyriales. An obligate mutualism with the fungi found in carton galleries of *Azteca brevis* is found for the host-ant with the ascomycete order Chaetothyriales; on the level of fungal species, no obligate mutualism is found. Moreover, *Azteca brevis* does not seem to strongly select for a particular morphological type, as both hyaline, thin-walled hyphae and pigmented, thick-walled hyphae are present in the carton. *Azteca brevis* cultivates and uses many different kinds of Chaetothyriales, and future research is needed to clarify the origins of these fungi. The reasons for the general preference of black yeasts in such ant–plant–fungus associations are still unclear. Knowledge of the diversity, coevolutionary processes and functional role of fungi in ant–plant symbioses is currently very fragmentary and further investigation is needed.

## Supporting Information

Appendix S1 **File contains Figures S1 and S2 and Tables S1–S4.**
(DOCX)
