# Supplementary material for: High Diversity and Low Specificity of Chaetothyrialean Fungi in Carton Galleries in a Neotropical Ant–Plant Association
Source: PLoS One. 2014 Nov 14;9(11):e112756. doi: 10.1371/journal.pone.0112756 (PMC4232418; doi:10.1371/journal.pone.0112756)
Supplement: Appendix S1 — File contains Figures S1 and S2 and Tables S1–S4. (DOCX) [file pone.0112756.s001.docx]

# Supplementary information

# High Biodiversity and Low Specificity of Chaetothyrialean Fungi on Carton Galleries in a Neotropical Ant–Plant Association

## Maximilian Nepel, Hermann Voglmayr, Jürg Schönenberger, Veronika E. Mayer

## PCR

The total PCR volume of 14 μL consisted of 6.3 µL 2.0× ReddyMix Extensor PCR Reddy Master Mix, 3.5 µL 1.1× ReddyMix PCR Master Mix, 0.45 µL of each primer (see Table S1), 2.6 µL ddH_2_O and 0.7 µL DNA extract. The PCR protocol was: 2 min denaturation at 94 °C; 15 cycles of 15 s denaturation at 94 °C, 30 s annealing at 53 °C, 1 min 50 s extension at 70 °C; 20 further cycles, as before except that extension lasted 2 min in each cycle; 3 min at 70 °C as final extension.

## Primer sequences

Table S1. Sequence and source of primers used for PCR and sequencing.

| Primer | Sequence (5′ to 3′) | Source |
| --- | --- | --- |
| F5.8Sr | TGC GTT CAA ARA TTC GAT G | Jaklitsch & Voglmayr (2011) |
| F5.8Sf | CAA CAA CGG ATC TCT TGG YTC | Jaklitsch & Voglmayr (2011) |
| ITS4 | TCC TCC GCT TAT TGA TAT GC | White *et al.* (1990) |
| LR2R-A | CAG AGA CCG ATA GCG CAC | Voglmayr *et al.* (2012) |
| LR2-A | TGC TTT TCA TCT TTC GAT CAC | Voglmayr *et al.* (2012) |
| LR3-CH | GGT ATA GGG GCG AAA GAC TAA TC | this study |
| LR3 | CCG TGT TTC AAG ACG GG | Vilgalys & Hester (1990) |
| LR5 | TCC TGA GGG AAA CTT CG | Vilgalys & Hester (1990) |
| V9G | TTA AGT CCC TGC CCT TTG TA | De Hoog & Gerrits van den Ende (1998) |

## Sequencing

Each sequencing reaction contained 2 µL PCR product, 0.5 µL BigDye, 2 µL 5× sequencing buffer, 0.3 µL primer (10 µM) and 5.2 µL ddH_2_O. The cycle-sequencing protocol consisted of an initial 1-min denaturation at 96 °C, followed by 40 cycles of 10 s denaturation at 96 °C, 5 s annealing at 50 °C and 3 min extension at 60 °C.

**
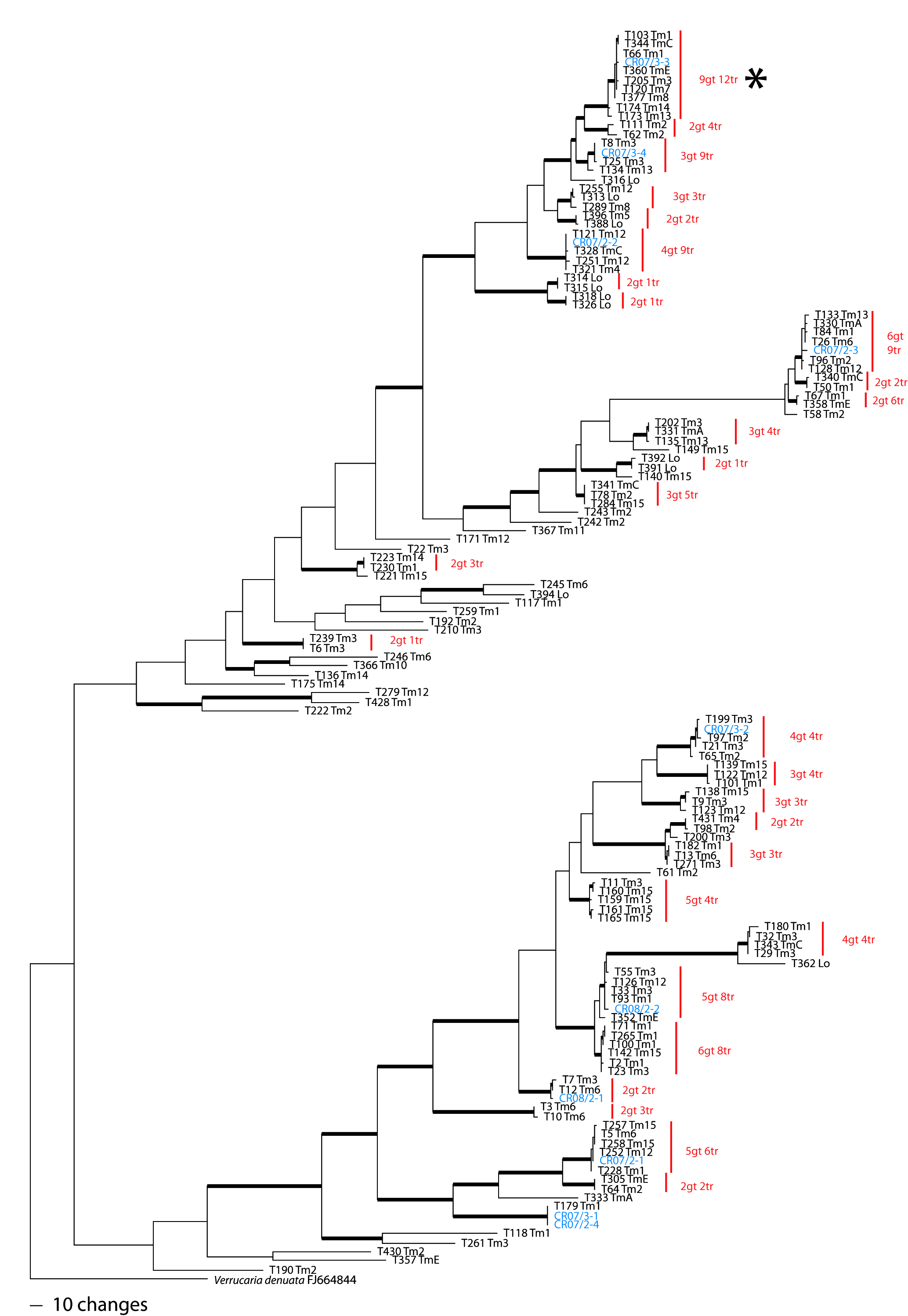
**Figure S1. Chaetothyrialean genotypes associated with *Azteca brevis* combined into operational taxonomic units (OTUs). The reduced phylogenetic tree of Chaetothyriales is based on the best ML search tree, using partial SSU, complete ITS and 5.8S, and partial LSU regions of carton fungal genotypes associated with *Azteca brevis*. Bold branches are supported by BA probabilities higher than 90%. Blue labels denote carton fungal genotypes sequenced and first published by Mayer & Voglmayr (2009); genotypes with fewer than 13 mutations are merged into OTUs, shown in red type; the mutation limit was calcu­lated from the average sequence length and the maximum intraspecific variation of the ITS region (Schoch *et al.* 2012); every OTU is represented by a number of geno­types (gt) and appears on a number of trees (tr). Note that after merging similar genotypes to get species-like classifications, the most common OTU can be found on 12 out of 19 trees (labelled with asterisk, *).


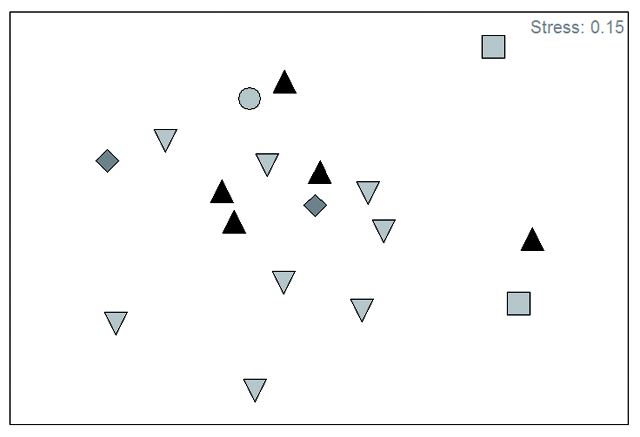


Figure S2. Correlation analysis of the fungal community at approximate species level against collection site. Non-metric multidimensional scaling (NMDS) plot based on Bray–Curtis similarities between sets of fungal OTUs occurring on carton material of sampled *Tetrathylacium macrophyllum* trees, including *Lonchocarpus* sp., colonised by *Azteca brevis* ants. Symbols represent trees of the same collection site (squares: Waterfall; triangles: Bird Trail; circle: Río Gamba; diamonds: Río Bolsa; inverted triangle: Río Sardinal). Note the diffuse arrangement indicating no correlation between set of OTUs and collection site.

Table S2. List of fungal genotypes. All genotypes belonging to the order Chaetothyriales isolated from carton build by *Azteca brevis* on two different plant species are listed, with GenBank accession numbers for the SSU–ITS–LSU locus. Carton samples were collected on different plant specimens (tree ID) near the Biological Research Station La Gamba, Golfito, southern Puntarenas, Costa Rica.

| Genotype | Plant species | GenBank accession number | Collection site | Tree ID | Longitude | Latitude | Collection date |
| --- | --- | --- | --- | --- | --- | --- | --- |
| T2 | *Tetrathylacium macrophyllum* | KF614798 | Bird Trail | Tm1 | 83° 12′ 14″ W | 8° 41′ 53″ N | February 2011 |
| T3 | *T. macrophyllum* | KF614769 | Río Sardinal | Tm6 | 83° 12′ 47″ W | 8° 43′ 29″ N | February 2011 |
| T5 | *T. macrophyllum* | KF614817 | Río Sardinal | Tm6 | 83° 12′ 47″ W | 8° 43′ 29″ N | February 2011 |
| T6 | *T. macrophyllum* | KF614883 | Bird Trail | Tm3 | 83° 12′ 15″ W | 8° 41′ 51″ N | February 2011 |
| T7 | *T. macrophyllum* | KF614794 | Bird Trail | Tm3 | 83° 12′ 15″ W | 8° 41′ 51″ N | February 2011 |
| T8 | *T. macrophyllum* | KF614824 | Bird Trail | Tm3 | 83° 12′ 15″ W | 8° 41′ 51″ N | February 2011 |
| T9 | *T. macrophyllum* | KF614780 | Bird Trail | Tm3 | 83° 12′ 15″ W | 8° 41′ 51″ N | February 2011 |
| T10 | *T. macrophyllum* | KF614770 | Río Sardinal | Tm6 | 83° 12′ 47″ W | 8° 43′ 29″ N | February 2011 |
| T11 | *T. macrophyllum* | KF614788 | Bird Trail | Tm3 | 83° 12′ 15″ W | 8° 41′ 51″ N | February 2011 |
| T12 | *T. macrophyllum* | KF614795 | Río Sardinal | Tm6 | 83° 12′ 47″ W | 8° 43′ 29″ N | February 2011 |
| T13 | *T. macrophyllum* | KF614778 | Río Sardinal | Tm6 | 83° 12′ 47″ W | 8° 43′ 29″ N | February 2011 |
| T21 | *T. macrophyllum* | KF614787 | Bird Trail | Tm3 | 83° 12′ 15″ W | 8° 41′ 51″ N | February 2011 |
| T22 | *T. macrophyllum* | KF614881 | Bird Trail | Tm3 | 83° 12′ 15″ W | 8° 41′ 51″ N | February 2011 |
| T23 | *T. macrophyllum* | KF614802 | Bird Trail | Tm3 | 83° 12′ 15″ W | 8° 41′ 51″ N | February 2011 |
| T25 | *T. macrophyllum* | KF614823 | Bird Trail | Tm3 | 83° 12′ 15″ W | 8° 41′ 51″ N | February 2011 |
| T26 | *T. macrophyllum* | KF614863 | Río Sardinal | Tm6 | 83° 12′ 47″ W | 8° 43′ 29″ N | February 2011 |
| T29 | *T. macrophyllum* | KF614810 | Bird Trail | Tm3 | 83° 12′ 15″ W | 8° 41′ 51″ N | February 2011 |
| T32 | *T. macrophyllum* | KF614807 | Bird Trail | Tm3 | 83° 12′ 15″ W | 8° 41′ 51″ N | February 2011 |
| T33 | *T. macrophyllum* | KF614801 | Bird Trail | Tm3 | 83° 12′ 15″ W | 8° 41′ 51″ N | February 2011 |
| T50 | *T. macrophyllum* | KF614857 | Bird Trail | Tm1 | 83° 12′ 14″ W | 8° 41′ 53″ N | February 2011 |
| T55 | *T. macrophyllum* | KF614808 | Bird Trail | Tm3 | 83° 12′ 15″ W | 8° 41′ 51″ N | February 2011 |
| T58 | *T. macrophyllum* | KF614870 | Bird Trail | Tm2 | 83° 12′ 14″ W | 8° 41′ 52″ N | February 2011 |
| T61 | *T. macrophyllum* | KF614783 | Bird Trail | Tm2 | 83° 12′ 14″ W | 8° 41′ 52″ N | February 2011 |
| T62 | *T. macrophyllum* | KF614826 | Bird Trail | Tm2 | 83° 12′ 14″ W | 8° 41′ 52″ N | February 2011 |
| T64 | *T. macrophyllum* | KF614815 | Bird Trail | Tm2 | 83° 12′ 14″ W | 8° 41′ 52″ N | February 2011 |
| T65 | *T. macrophyllum* | KF614786 | Bird Trail | Tm2 | 83° 12′ 14″ W | 8° 41′ 52″ N | February 2011 |
| T66 | *T. macrophyllum* | KF614834 | Bird Trail | Tm1 | 83° 12′ 14″ W | 8° 41′ 53″ N | February 2011 |
| T67 | *T. macrophyllum* | KF614867 | Bird Trail | Tm1 | 83° 12′ 14″ W | 8° 41′ 53″ N | February 2011 |
| T71 | *T. macrophyllum* | KF614796 | Bird Trail | Tm1 | 83° 12′ 14″ W | 8° 41′ 53″ N | February 2011 |
| T78 | *T. macrophyllum* | KF614865 | Bird Trail | Tm2 | 83° 12′ 14″ W | 8° 41′ 52″ N | February 2011 |
| T84 | *T. macrophyllum* | KF614860 | Bird Trail | Tm1 | 83° 12′ 14″ W | 8° 41′ 53″ N | February 2011 |
| T93 | *T. macrophyllum* | KF614799 | Bird Trail | Tm1 | 83° 12′ 14″ W | 8° 41′ 53″ N | February 2011 |
| T96 | *T. macrophyllum* | KF614861 | Bird Trail | Tm2 | 83° 12′ 14″ W | 8° 41′ 52″ N | February 2011 |
| T97 | *T. macrophyllum* | KF614784 | Bird Trail | Tm2 | 83° 12′ 14″ W | 8° 41′ 52″ N | February 2011 |
| T98 | *T. macrophyllum* | KF614774 | Bird Trail | Tm2 | 83° 12′ 14″ W | 8° 41′ 52″ N | February 2011 |
| T100 | *T. macrophyllum* | KF614803 | Bird Trail | Tm1 | 83° 12′ 14″ W | 8° 41′ 53″ N | February 2011 |
| T101 | *T. macrophyllum* | KF614772 | Bird Trail | Tm1 | 83° 12′ 14″ W | 8° 41′ 53″ N | February 2011 |
| T103 | *T. macrophyllum* | KF614829 | Bird Trail | Tm1 | 83° 12′ 14″ W | 8° 41′ 53″ N | February 2011 |
| T111 | *T. macrophyllum* | KF614825 | Bird Trail | Tm2 | 83° 12′ 14″ W | 8° 41′ 52″ N | February 2011 |
| T117 | *T. macrophyllum* | KF614891 | Bird Trail | Tm1 | 83° 12′ 14″ W | 8° 41′ 53″ N | February 2011 |
| T118 | *T. macrophyllum* | KF614811 | Bird Trail | Tm1 | 83° 12′ 14″ W | 8° 41′ 53″ N | February 2011 |
| T120 | *T. macrophyllum* | KF614832 | Río Sardinal | Tm7 | 83° 13′ 0″ W | 8° 43′ 66″ N | February 2011 |
| T121 | *T. macrophyllum* | KF614848 | Río Sardinal | Tm12 | 83° 13′ 28″ W | 8° 43′ 48″ N | February 2011 |
| T122 | *T. macrophyllum* | KF614782 | Río Sardinal | Tm12 | 83° 13′ 28″ W | 8° 43′ 48″ N | February 2011 |
| T123 | *T. macrophyllum* | KF614779 | Río Sardinal | Tm12 | 83° 13′ 28″ W | 8° 43′ 48″ N | February 2011 |
| T126 | *T. macrophyllum* | KF614800 | Río Sardinal | Tm12 | 83° 13′ 28″ W | 8° 43′ 48″ N | February 2011 |
| T128 | *T. macrophyllum* | KF614862 | Río Sardinal | Tm12 | 83° 13′ 28″ W | 8° 43′ 48″ N | February 2011 |
| T133 | *T. macrophyllum* | KF614858 | Río Sardinal | Tm13 | 83° 13′ 30″ W | 8° 43′ 53″ N | February 2011 |
| T134 | *T. macrophyllum* | KF614822 | Río Sardinal | Tm13 | 83° 13′ 30″ W | 8° 43′ 53″ N | February 2011 |
| T135 | *T. macrophyllum* | KF614853 | Río Sardinal | Tm13 | 83° 13′ 30″ W | 8° 43′ 53″ N | February 2011 |
| T136 | *T. macrophyllum* | KF614890 | Río Sardinal | Tm14 | 83° 13′ 31″ W | 8° 43′ 59″ N | February 2011 |
| T138 | *T. macrophyllum* | KF614771 | Río Sardinal | Tm15 | 83° 13′ 31″ W | 8° 44′ 0″ N | February 2011 |
| T139 | *T. macrophyllum* | KF614781 | Río Sardinal | Tm15 | 83° 13′ 31″ W | 8° 44′ 0″ N | February 2011 |
| T140 | *T. macrophyllum* | KF614850 | Río Sardinal | Tm15 | 83° 13′ 31″ W | 8° 44′ 0″ N | February 2011 |
| T142 | *T. macrophyllum* | KF614797 | Río Sardinal | Tm15 | 83° 13′ 31″ W | 8° 44′ 0″ N | February 2011 |
| T149 | *T. macrophyllum* | KF614849 | Río Sardinal | Tm15 | 83° 13′ 31″ W | 8° 44′ 0″ N | February 2011 |
| T159 | *T. macrophyllum* | KF614790 | Río Sardinal | Tm15 | 83° 13′ 31″ W | 8° 44′ 0″ N | February 2011 |
| T160 | *T. macrophyllum* | KF614789 | Río Sardinal | Tm15 | 83° 13′ 31″ W | 8° 44′ 0″ N | February 2011 |
| T161 | *T. macrophyllum* | KF614791 | Río Sardinal | Tm15 | 83° 13′ 31″ W | 8° 44′ 0″ N | February 2011 |
| T165 | *T. macrophyllum* | KF614792 | Río Sardinal | Tm15 | 83° 13′ 31″ W | 8° 44′ 0″ N | February 2011 |
| T171 | *T. macrophyllum* | KF614875 | Río Sardinal | Tm12 | 83° 13′ 28″ W | 8° 43′ 48″ N | February 2011 |
| T173 | *T. macrophyllum* | KF614827 | Río Sardinal | Tm13 | 83° 13′ 30″ W | 8° 43′ 53″ N | February 2011 |
| T174 | *T. macrophyllum* | KF614828 | Río Sardinal | Tm14 | 83° 13′ 31″ W | 8° 43′ 59″ N | February 2011 |
| T175 | *T. macrophyllum* | KF614879 | Río Sardinal | Tm14 | 83° 13′ 31″ W | 8° 43′ 59″ N | February 2011 |
| T179 | *T. macrophyllum* | KF614876 | Bird Trail | Tm1 | 83° 12′ 14″ W | 8° 41′ 53″ N | February 2011 |
| T180 | *T. macrophyllum* | KF614806 | Bird Trail | Tm1 | 83° 12′ 14″ W | 8° 41′ 53″ N | February 2011 |
| T182 | *T. macrophyllum* | KF614776 | Bird Trail | Tm1 | 83° 12′ 14″ W | 8° 41′ 53″ N | February 2011 |
| T190 | *T. macrophyllum* | KF614874 | Bird Trail | Tm2 | 83° 12′ 14″ W | 8° 41′ 52″ N | February 2011 |
| T192 | *T. macrophyllum* | KF614877 | Bird Trail | Tm2 | 83° 12′ 14″ W | 8° 41′ 52″ N | February 2011 |
| T199 | *T. macrophyllum* | KF614785 | Bird Trail | Tm3 | 83° 12′ 15″ W | 8° 41′ 51″ N | February 2011 |
| T200 | *T. macrophyllum* | KF614775 | Bird Trail | Tm3 | 83° 12′ 15″ W | 8° 41′ 51″ N | February 2011 |
| T202 | *T. macrophyllum* | KF614854 | Bird Trail | Tm3 | 83° 12′ 15″ W | 8° 41′ 51″ N | February 2011 |
| T205 | *T. macrophyllum* | KF614835 | Bird Trail | Tm3 | 83° 12′ 15″ W | 8° 41′ 51″ N | February 2011 |
| T210 | *T. macrophyllum* | KF614880 | Bird Trail | Tm3 | 83° 12′ 15″ W | 8° 41′ 51″ N | February 2011 |
| T221 | *T. macrophyllum* | KF614892 | Río Sardinal | Tm15 | 83° 13′ 31″ W | 8° 44′ 0″ N | February 2011 |
| T222 | *T. macrophyllum* | KF614812 | Bird Trail | Tm2 | 83° 12′ 14″ W | 8° 41′ 52″ N | February 2011 |
| T223 | *T. macrophyllum* | KF614884 | Río Sardinal | Tm14 | 83° 13′ 31″ W | 8° 43′ 59″ N | February 2011 |
| T228 | *T. macrophyllum* | KF614820 | Bird Trail | Tm1 | 83° 12′ 14″ W | 8° 41′ 53″ N | February 2011 |
| T230 | *T. macrophyllum* | KF614885 | Bird Trail | Tm1 | 83° 12′ 14″ W | 8° 41′ 53″ N | February 2011 |
| T239 | *T. macrophyllum* | KF614882 | Bird Trail | Tm3 | 83° 12′ 15″ W | 8° 41′ 51″ N | February 2011 |
| T242 | *T. macrophyllum* | KF614869 | Bird Trail | Tm2 | 83° 12′ 14″ W | 8° 41′ 52″ N | February 2011 |
| T243 | *T. macrophyllum* | KF614871 | Bird Trail | Tm2 | 83° 12′ 14″ W | 8° 41′ 52″ N | February 2011 |
| T245 | *T. macrophyllum* | KF614893 | Río Sardinal | Tm6 | 83° 12′ 47″ W | 8° 43′ 29″ N | February 2011 |
| T246 | *T. macrophyllum* | KF614878 | Río Sardinal | Tm6 | 83° 12′ 47″ W | 8° 43′ 29″ N | February 2011 |
| T251 | *T. macrophyllum* | KF614845 | Río Sardinal | Tm12 | 83° 13′ 28″ W | 8° 43′ 48″ N | February 2011 |
| T252 | *T. macrophyllum* | KF614819 | Río Sardinal | Tm12 | 83° 13′ 28″ W | 8° 43′ 48″ N | February 2011 |
| T255 | *T. macrophyllum* | KF614838 | Río Sardinal | Tm12 | 83° 13′ 28″ W | 8° 43′ 48″ N | February 2011 |
| T257 | *T. macrophyllum* | KF614816 | Río Sardinal | Tm15 | 83° 13′ 31″ W | 8° 44′ 0″ N | February 2011 |
| T258 | *T. macrophyllum* | KF614818 | Río Sardinal | Tm15 | 83° 13′ 31″ W | 8° 44′ 0″ N | February 2011 |
| T259 | *T. macrophyllum* | KF614887 | Bird Trail | Tm1 | 83° 12′ 14″ W | 8° 41′ 53″ N | February 2011 |
| T261 | *T. macrophyllum* | KF614886 | Bird Trail | Tm3 | 83° 12′ 15″ W | 8° 41′ 51″ N | February 2011 |
| T265 | *T. macrophyllum* | KF614804 | Bird Trail | Tm1 | 83° 12′ 14″ W | 8° 41′ 53″ N | February 2011 |
| T271 | *T. macrophyllum* | KF614777 | Bird Trail | Tm3 | 83° 12′ 15″ W | 8° 41′ 51″ N | February 2011 |
| T279 | *T. macrophyllum* | KF614767 | Río Sardinal | Tm12 | 83° 13′ 28″ W | 8° 43′ 48″ N | February 2011 |
| T284 | *T. macrophyllum* | KF614866 | Río Sardinal | Tm15 | 83° 13′ 31″ W | 8° 44′ 0″ N | February 2011 |
| T289 | *T. macrophyllum* | KF614840 | Río Sardinal | Tm8 | 83° 13′ 12″ W | 8° 43′ 49″ N | February 2011 |
| T305 | *T. macrophyllum* | KF614814 | Río Gamba | TmE | 83° 11′ 53″ W | 8° 40′ 47″ N | January 2009 |
| T321 | *T. macrophyllum* | KF614847 | Bird Trail | Tm4 | 83° 12′ 16″ W | 8° 41′ 50″ N | February 2011 |
| T328 | *T. macrophyllum* | KF614846 | Río Bolsa | TmC | 83° 10′ 35″ W | 8° 41′ 1″ N | January 2009 |
| T330 | *T. macrophyllum* | KF614859 | Río Bolsa | TmA | 83° 10′ 35″ W | 8° 41′ 6″ N | January 2009 |
| T331 | *T. macrophyllum* | KF614855 | Río Bolsa | TmA | 83° 10′ 35″ W | 8° 41′ 6″ N | January 2009 |
| T333 | *T. macrophyllum* | KF614873 | Río Bolsa | TmA | 83° 10′ 35″ W | 8° 41′ 6″ N | January 2009 |
| T340 | *T. macrophyllum* | KF614856 | Río Bolsa | TmC | 83° 10′ 35″ W | 8° 41′ 1″ N | January 2009 |
| T341 | *T. macrophyllum* | KF614864 | Río Bolsa | TmC | 83° 10′ 35″ W | 8° 41′ 1″ N | January 2009 |
| T343 | *T. macrophyllum* | KF614809 | Río Bolsa | TmC | 83° 10′ 35″ W | 8° 41′ 1″ N | January 2009 |
| T344 | *T. macrophyllum* | KF614830 | Río Bolsa | TmC | 83° 10′ 35″ W | 8° 41′ 1″ N | January 2009 |
| T352 | *T. macrophyllum* | KF614805 | Río Gamba | TmE | 83° 11′ 53″ W | 8° 40′ 47″ N | January 2009 |
| T357 | *T. macrophyllum* | KF614872 | Río Gamba | TmE | 83° 11′ 53″ W | 8° 40′ 47″ N | January 2009 |
| T358 | *T. macrophyllum* | KF614868 | Río Gamba | TmE | 83° 11′ 53″ W | 8° 40′ 47″ N | January 2009 |
| T360 | *T. macrophyllum* | KF614833 | Río Gamba | TmE | 83° 11′ 53″ W | 8° 40′ 47″ N | January 2009 |
| T366 | *T. macrophyllum* | KF614888 | Waterfall | Tm10 | 83° 10′ 28″ W | 8° 42′ 23″ N | February 2011 |
| T3671 | *T. macrophyllum* | KF614894 | Waterfall | Tm11 | 83° 10′ 26″ W | 8° 42′ 23″ N | February 2011 |
| T377 | *T. macrophyllum* | KF614831 | Río Sardinal | Tm8 | 83° 13′ 12″ W | 8° 43′ 49″ N | February 2011 |
| T396 | *T. macrophyllum* | KF614837 | Bird Trail | Tm5 | 83° 12′ 18″ W | 8° 41′ 49″ N | February 2011 |
| T428 | *T. macrophyllum* | KF614768 | Bird Trail | Tm1 | 83° 12′ 14″ W | 8° 41′ 53″ N | February 2011 |
| T430 | *T. macrophyllum* | KF614813 | Bird Trail | Tm2 | 83° 12′ 14″ W | 8° 41′ 52″ N | February 2011 |
| T431 | *T. macrophyllum* | KF614773 | Bird Trail | Tm4 | 83° 12′ 16″ W | 8° 41′ 50″ N | February 2011 |
| T313 | *Lonchocarpus sp.* | KF614839 | Waterfall | Lo | 83° 10′ 27″ W | 8° 42′ 23″ N | February 2011 |
| T314 | *Lonchocarpus sp.* | KF614841 | Waterfall | Lo | 83° 10′ 27″ W | 8° 42′ 23″ N | February 2011 |
| T315 | *Lonchocarpus sp.* | KF614842 | Waterfall | Lo | 83° 10′ 27″ W | 8° 42′ 23″ N | February 2011 |
| T316 | *Lonchocarpus sp.* | KF614821 | Waterfall | Lo | 83° 10′ 27″ W | 8° 42′ 23″ N | February 2011 |
| T318 | *Lonchocarpus sp.* | KF614843 | Waterfall | Lo | 83° 10′ 27″ W | 8° 42′ 23″ N | February 2011 |
| T326 | *Lonchocarpus sp.* | KF614844 | Waterfall | Lo | 83° 10′ 27″ W | 8° 42′ 23″ N | February 2011 |
| T362 | *Lonchocarpus sp.* | KF614793 | Waterfall | Lo | 83° 10′ 27″ W | 8° 42′ 23″ N | February 2011 |
| T388 | *Lonchocarpus sp.* | KF614836 | Waterfall | Lo | 83° 10′ 27″ W | 8° 42′ 23″ N | February 2011 |
| T391 | *Lonchocarpus sp.* | KF614852 | Waterfall | Lo | 83° 10′ 27″ W | 8° 42′ 23″ N | February 2011 |
| T392 | *Lonchocarpus sp.* | KF614851 | Waterfall | Lo | 83° 10′ 27″ W | 8° 42′ 23″ N | February 2011 |
| T394 | *Lonchocarpus sp.* | KF614889 | Waterfall | Lo | 83° 10′ 27″ W | 8° 42′ 23″ N | February 2011 |

Table S3. Presence–absence matrix of carton fungal genotypes on trees colonised by *Azteca brevis*. Presence–absence matrix for 128 genotypes of carton fungi on 19 trees (Tm: *Tetrathylacium macrophyllum*, Lo: *Lonchocarpus* sp.) at five collection sites. “Seq ID” is the sequence number in the phylogenetic tree (Figure 2-3) representing one genotype.

| Geno­type | Bird Trail – Lodge | | | | |  | Río Sardinal | | | |  | Waterfall | |  | Río Sardinal | | | |  | Río Bolsa | |  | Río Gamba |  | Waterfall | Seq ID |
| --- | --- | --- | --- | --- | --- | --- | --- | --- | --- | --- | --- | --- | --- | --- | --- | --- | --- | --- | --- | --- | --- | --- | --- | --- | --- | --- |
|  | Tm1 | Tm2 | Tm3 | Tm4 | Tm5 |  | Tm6 | Tm7 | Tm8 | Tm9 |  | Tm10 | Tm11 |  | Tm12 | Tm13 | Tm14 | Tm15 |  | TmA | TmC |  | TmE |  | Lo |  |
| 1 | 1 | 1 | 1 | 1 |  |  |  |  |  |  |  |  |  |  | 1 |  |  |  |  |  |  |  |  |  |  | T66 |
| 2 | 1 |  |  |  |  |  |  |  | 1 | 1 |  |  |  |  |  |  |  |  |  |  |  |  |  |  |  | T103 |
| 3 |  |  |  |  |  |  |  |  |  |  |  |  |  |  |  |  |  |  |  |  | 1 |  |  |  |  | T344 |
| 4 |  |  |  |  |  |  |  | 1 |  |  |  |  |  |  |  |  |  |  |  |  |  |  |  |  |  | T120 |
| 5 |  |  | 1 |  |  |  |  |  |  |  |  |  |  |  |  |  |  |  |  |  |  |  |  |  |  | T205 |
| 6 |  |  |  |  |  |  |  |  |  |  |  |  |  |  |  |  |  |  |  |  |  |  | 1 |  |  | T360 |
| 7 |  |  |  |  |  |  |  |  | 1 |  |  |  |  |  |  |  |  |  |  |  |  |  |  |  |  | T377 |
| 8 |  |  |  |  |  |  |  |  |  |  |  |  |  |  |  |  | 1 |  |  |  |  |  |  |  |  | T174 |
| 9 |  |  |  |  |  |  |  |  |  |  |  |  |  |  |  | 1 |  |  |  |  |  |  |  |  |  | T173 |
| 10 |  | 1 |  | 1 |  |  |  |  | 1 | 1 |  |  |  |  |  |  |  |  |  |  |  |  |  |  |  | T111 |
| 11 |  | 1 |  |  |  |  |  |  |  |  |  |  |  |  |  |  |  |  |  |  |  |  |  |  |  | T62 |
| 12 | 1 |  | 1 |  |  |  |  |  |  |  |  |  |  |  |  |  |  |  |  |  |  |  |  |  |  | T25 |
| 13 | 1 | 1 | 1 | 1 |  |  |  |  |  | 1 |  |  |  |  |  |  |  |  |  |  | 1 |  |  |  | 1 | T8 |
| 14 |  |  |  |  |  |  |  |  | 1 |  |  |  |  |  |  | 1 |  |  |  |  |  |  |  |  |  | T134 |
| 15 |  |  |  |  |  |  |  |  |  |  |  |  |  |  |  |  |  |  |  |  |  |  |  |  | 1 | T316 |
| 16 |  |  |  |  |  |  |  |  |  |  |  |  |  |  | 1 |  |  |  |  |  |  |  |  |  |  | T251 |
| 17 |  |  |  |  |  |  |  |  |  |  |  |  |  |  |  |  |  |  |  |  | 1 |  |  |  |  | T328 |
| 18 |  |  | 1 | 1 | 1 |  |  | 1 | 1 | 1 |  |  | 1 |  | 1 |  |  |  |  |  | 1 |  |  |  |  | T121 |
| 19 |  |  |  | 1 |  |  |  |  |  |  |  |  |  |  |  |  |  |  |  |  |  |  |  |  |  | T321 |
| 20 |  |  |  |  |  |  |  |  |  |  |  |  |  |  | 1 |  |  |  |  |  |  |  |  |  |  | T255 |
| 21 |  |  |  |  |  |  |  |  |  |  |  |  |  |  |  |  |  |  |  |  |  |  |  |  | 1 | T313 |
| 22 |  |  |  |  |  |  |  |  | 1 |  |  |  |  |  |  |  |  |  |  |  |  |  |  |  |  | T289 |
| 23 |  |  |  |  |  |  |  |  |  |  |  |  |  |  |  |  |  |  |  |  |  |  |  |  | 1 | T388 |
| 24 |  |  |  |  | 1 |  |  |  |  |  |  |  |  |  |  |  |  |  |  |  |  |  |  |  |  | T396 |
| 25 |  |  |  |  |  |  |  |  |  |  |  |  |  |  |  |  |  |  |  |  |  |  |  |  | 1 | T318 |
| 26 |  |  |  |  |  |  |  |  |  |  |  |  |  |  |  |  |  |  |  |  |  |  |  |  | 1 | T326 |
| 27 |  |  |  |  |  |  |  |  |  |  |  |  |  |  |  |  |  |  |  |  |  |  |  |  | 1 | T314 |
| 28 |  |  |  |  |  |  |  |  |  |  |  |  |  |  |  |  |  |  |  |  |  |  |  |  | 1 | T315 |
| 29 |  |  |  |  |  |  |  |  |  |  |  |  |  |  |  |  |  |  |  | 1 |  |  |  |  |  | T331 |
| 30 |  |  |  |  |  |  |  |  |  |  |  |  |  |  |  | 1 |  |  |  |  |  |  |  |  |  | T135 |
| 31 |  |  | 1 | 1 |  |  |  |  |  |  |  |  |  |  |  |  |  |  |  |  |  |  |  |  |  | T202 |
| 32 |  |  |  |  |  |  |  |  |  |  |  |  |  |  |  |  |  | 1 |  |  |  |  |  |  |  | T149 |
| 33 |  | 1 |  | 1 |  |  |  |  |  |  |  |  |  |  |  |  |  |  |  |  |  |  |  |  |  | T78 |
| 34 |  |  |  |  |  |  |  |  |  |  |  |  |  |  |  |  |  | 1 |  | 1 |  |  |  |  |  | T284 |
| 35 |  |  |  |  |  |  |  |  |  |  |  |  |  |  |  |  |  |  |  |  | 1 |  |  |  |  | T341 |
| 36 |  |  |  |  |  |  |  |  |  |  |  |  |  |  |  |  |  |  |  |  |  |  |  |  | 1 | T392 |
| 37 |  |  |  |  |  |  |  |  |  |  |  |  |  |  |  |  |  |  |  |  |  |  |  |  | 1 | T391 |
| 38 |  |  |  |  |  |  |  |  |  |  |  |  |  |  |  |  |  | 1 |  |  |  |  |  |  |  | T140 |
| 39 | 1 |  |  |  |  |  |  |  |  |  |  |  |  |  |  |  |  |  |  |  |  |  |  |  |  | TT67 |
| 40 | 1 | 1 | 1 |  |  |  |  |  | 1 | 1 |  |  |  |  |  |  |  |  |  |  |  |  | 1 |  |  | T358 |
| 41 |  | 1 |  |  |  |  |  |  |  |  |  |  |  |  |  |  |  |  |  |  |  |  |  |  |  | T58 |
| 42 | 1 |  |  |  |  |  |  |  | 1 |  |  |  |  |  |  |  |  | 1 |  |  | 1 |  |  |  |  | T84 |
| 43 |  | 1 |  |  |  |  |  |  |  |  |  |  |  |  |  |  |  | 1 |  |  |  |  |  |  |  | T96 |
| 44 |  |  |  |  |  |  |  |  |  |  |  |  |  |  |  |  |  |  |  | 1 |  |  |  |  |  | T330 |
| 45 |  | 1 |  |  |  |  | 1 |  |  |  |  |  |  |  |  |  |  |  |  |  |  |  |  |  |  | T26 |
| 46 |  |  |  |  |  |  |  |  |  |  |  |  |  |  | 1 |  |  |  |  |  |  |  |  |  |  | T128 |
| 47 |  |  |  |  |  |  |  |  |  |  |  |  |  |  |  | 1 |  |  |  |  |  |  |  |  |  | T133 |
| 48 |  |  |  |  |  |  |  |  |  |  |  |  |  |  |  |  |  |  |  |  | 1 |  |  |  |  | T340 |
| 49 | 1 |  |  |  |  |  |  |  |  |  |  |  |  |  |  |  |  |  |  |  | 1 |  |  |  |  | T50 |
| 50 |  | 1 |  |  |  |  |  |  |  |  |  |  |  |  |  |  |  |  |  |  |  |  |  |  |  | T243 |
| 51 |  | 1 |  |  |  |  |  |  |  |  |  |  |  |  |  |  |  |  |  |  |  |  |  |  |  | T242 |
| 52 |  |  |  |  |  |  |  |  |  |  |  |  | 1 |  |  |  |  |  |  |  |  |  |  |  |  | T367 |
| 53 |  |  |  |  |  |  |  |  |  |  |  |  |  |  | 1 |  |  |  |  |  |  |  |  |  |  | T171 |
| 54 |  |  | 1 |  |  |  |  |  |  |  |  |  |  |  |  |  |  |  |  |  |  |  |  |  |  | T22 |
| 55 |  |  | 1 |  |  |  |  |  |  |  |  |  |  |  |  |  |  |  |  |  |  |  |  |  |  | T239 |
| 56 |  |  | 1 |  |  |  |  |  |  |  |  |  |  |  |  |  |  |  |  |  |  |  |  |  |  | T6 |
| 57 |  |  |  |  |  |  |  |  |  |  |  |  |  |  |  | 1 | 1 |  |  |  |  |  |  |  |  | T136 |
| 58 |  |  |  |  |  |  |  |  |  |  |  | 1 |  |  |  |  |  |  |  |  |  |  |  |  |  | T366 |
| 59 |  |  |  |  |  |  | 1 |  |  |  |  |  |  |  |  |  |  |  |  |  |  |  |  |  |  | T246 |
| 60 |  |  | 1 |  |  |  |  |  | 1 |  |  |  |  |  |  |  |  |  |  |  |  |  |  |  |  | T210 |
| 61 |  |  | 1 |  |  |  |  |  |  |  |  |  |  |  |  |  | 1 |  |  |  |  |  |  |  |  | T223 |
| 62 | 1 |  |  |  |  |  |  |  |  |  |  |  |  |  |  |  |  |  |  |  |  |  |  |  |  | T230 |
| 63 |  |  |  |  |  |  | 1 |  |  |  |  |  |  |  |  |  | 1 | 1 |  |  |  |  |  |  |  | T221 |
| 64 | 1 |  |  |  |  |  |  |  |  |  |  |  |  |  |  |  |  |  |  |  |  |  |  |  |  | T259 |
| 65 | 1 |  |  |  |  |  |  |  |  |  |  |  |  |  |  |  |  |  |  |  |  |  |  |  |  | T117 |
| 66 |  |  |  |  |  |  |  |  |  |  |  |  |  |  |  |  |  |  |  |  |  |  |  |  | 1 | T394 |
| 67 |  |  |  |  |  |  | 1 |  |  |  |  |  |  |  |  |  |  |  |  |  |  |  |  |  |  | T245 |
| 68 |  |  |  |  |  |  |  |  |  |  |  |  |  |  |  |  | 1 |  |  |  |  |  |  |  |  | T175 |
| 69 |  | 1 |  |  |  |  |  |  |  |  |  |  |  |  |  |  |  |  |  |  |  |  |  |  |  | T192 |
| 70 |  | 1 |  |  |  |  |  |  |  |  |  |  |  |  |  |  |  |  |  |  |  |  |  |  |  | T222 |
| 71 |  |  |  |  |  |  |  |  |  |  |  |  |  |  | 1 |  |  |  |  |  |  |  |  |  |  | T279 |
| 72 | 1 |  |  |  |  |  |  |  |  |  |  |  |  |  |  |  |  |  |  |  |  |  |  |  |  | T428 |
| 73 |  | 1 |  |  |  |  |  |  |  |  |  |  |  |  |  |  |  |  |  |  |  |  |  |  |  | T190 |
| 74 |  | 1 |  |  |  |  |  |  |  |  |  |  |  |  |  |  |  |  |  |  |  |  |  |  |  | T430 |
| 75 |  |  |  |  |  |  |  |  |  |  |  |  |  |  |  |  |  |  |  |  |  |  | 1 |  |  | T357 |
| 76 |  | 1 |  |  |  |  |  |  |  |  |  |  |  |  |  |  |  | 1 |  |  |  |  |  |  |  | T97 |
| 77 |  |  | 1 |  |  |  |  |  |  |  |  |  |  |  |  |  |  |  |  |  |  |  |  |  |  | T199 |
| 78 |  |  | 1 |  |  |  |  |  |  |  |  |  |  |  |  |  |  |  |  |  |  |  |  |  |  | T21 |
| 79 | 1 | 1 |  |  |  |  |  |  |  |  |  |  |  |  |  |  |  |  |  |  |  |  |  |  |  | T65 |
| 80 | 1 |  |  |  |  |  |  |  |  |  |  |  |  |  |  |  |  |  |  |  |  |  |  |  |  | T101 |
| 81 |  |  |  |  |  |  |  |  |  |  |  |  |  |  |  |  |  | 1 |  |  |  |  |  |  |  | T139 |
| 82 |  |  |  |  |  |  |  |  |  |  |  |  |  |  | 1 |  |  |  |  |  |  |  | 1 |  |  | T122 |
| 83 |  | 1 |  |  |  |  |  |  |  |  |  |  |  |  |  |  |  |  |  |  |  |  |  |  |  | T61 |
| 84 | 1 |  | 1 |  |  |  |  |  |  |  |  |  |  |  |  |  |  |  |  |  |  |  |  |  |  | T11 |
| 85 |  |  |  | 1 |  |  |  |  |  |  |  |  |  |  |  |  |  | 1 |  |  |  |  |  |  |  | T160 |
| 86 |  |  |  |  |  |  |  |  |  |  |  |  |  |  |  |  |  | 1 |  |  |  |  |  |  |  | T161 |
| 87 |  |  |  |  |  |  |  |  |  |  |  |  |  |  |  |  |  | 1 |  |  |  |  |  |  |  | T165 |
| 88 |  |  |  |  |  |  |  |  |  |  |  |  |  |  |  |  |  | 1 |  |  |  |  |  |  |  | T159 |
| 89 |  |  | 1 |  |  |  |  |  |  |  |  |  |  |  |  |  |  |  |  |  |  |  |  |  |  | T7 |
| 90 |  |  |  |  |  |  | 1 |  |  |  |  |  |  |  |  |  |  |  |  |  |  |  |  |  |  | T12 |
| 91 |  |  | 1 |  |  |  |  |  |  |  |  |  |  |  |  |  |  |  |  |  |  |  |  |  |  | T9 |
| 92 |  |  |  |  |  |  |  |  |  |  |  |  |  |  |  |  |  | 1 |  |  |  |  |  |  |  | T138 |
| 93 |  |  |  |  |  |  |  |  |  |  |  |  |  |  | 1 |  |  |  |  |  |  |  |  |  |  | T123 |
| 94 | 1 |  |  |  |  |  |  |  |  |  |  |  |  |  |  |  |  |  |  |  |  |  |  |  |  | T182 |
| 95 |  |  |  |  |  |  | 1 |  |  |  |  |  |  |  |  |  |  |  |  |  |  |  |  |  |  | T13 |
| 96 |  |  | 1 |  |  |  |  |  |  |  |  |  |  |  |  |  |  |  |  |  |  |  |  |  |  | T271 |
| 97 |  |  | 1 |  |  |  |  |  |  |  |  |  |  |  |  |  |  |  |  |  |  |  |  |  |  | T200 |
| 98 |  |  |  | 1 |  |  |  |  |  |  |  |  |  |  |  |  |  |  |  |  |  |  |  |  |  | T431 |
| 99 |  | 1 |  |  |  |  |  |  |  |  |  |  |  |  |  |  |  |  |  |  |  |  |  |  |  | T98 |
| 100 | 1 |  |  |  |  |  |  |  |  |  |  |  |  |  |  |  |  |  |  |  |  |  |  |  |  | T71 |
| 101 | 1 |  |  | 1 |  |  |  |  |  |  |  |  |  |  |  |  |  |  |  |  |  |  |  |  |  | T265 |
| 102 | 1 |  |  |  |  |  |  |  |  |  |  |  |  |  |  |  |  |  |  |  |  |  |  |  |  | T100 |
| 103 |  |  |  | 1 |  |  |  |  |  |  |  |  |  |  |  |  |  | 1 |  |  |  |  |  |  |  | T142 |
| 104 | 1 |  |  |  |  |  |  |  |  |  |  |  |  |  |  |  |  |  |  |  |  |  |  |  |  | T2 |
| 105 | 1 | 1 | 1 |  |  |  |  |  |  |  |  |  |  |  | 1 |  |  |  |  | 1 |  |  | 1 |  |  | T23 |
| 106 |  |  |  |  |  |  |  |  |  |  |  |  |  |  | 1 |  |  | 1 |  |  |  |  |  |  |  | T126 |
| 107 | 1 |  |  |  |  |  |  |  |  |  |  |  |  |  |  |  |  |  |  |  |  |  |  |  |  | T93 |
| 108 | 1 | 1 | 1 | 1 |  |  |  |  |  |  |  |  |  |  | 1 |  |  | 1 |  |  | 1 |  |  |  |  | T33 |
| 109 |  |  | 1 |  |  |  |  |  |  |  |  |  |  |  |  |  |  |  |  |  |  |  |  |  |  | T55 |
| 110 |  |  |  |  |  |  |  |  |  |  |  |  |  |  |  |  |  |  |  |  |  |  | 1 |  |  | T352 |
| 111 | 1 |  |  |  |  |  |  |  |  |  |  |  |  |  |  |  |  |  |  |  |  |  |  |  |  | T180 |
| 112 |  |  | 1 |  |  |  |  |  |  |  |  |  |  |  |  |  |  |  |  |  |  |  |  |  |  | T32 |
| 113 |  |  |  |  |  |  |  |  |  |  |  |  |  |  |  |  |  |  |  |  | 1 |  |  |  |  | T343 |
| 114 | 1 |  | 1 |  |  |  |  |  |  |  |  |  |  |  |  |  |  | 1 |  |  | 1 |  |  |  |  | T29 |
| 115 |  |  |  |  |  |  |  |  |  |  |  |  |  |  |  |  |  |  |  |  |  |  |  |  | 1 | T362 |
| 116 |  |  | 1 |  |  |  | 1 | 1 |  |  |  |  |  |  |  |  |  |  |  |  |  |  |  |  |  | T10 |
| 117 |  |  |  |  |  |  | 1 |  |  |  |  |  |  |  |  |  |  |  |  |  |  |  |  |  |  | T3 |
| 118 |  |  |  |  |  |  |  |  |  |  |  |  |  |  |  |  |  | 1 |  |  |  |  |  |  |  | T257 |
| 119 |  |  | 1 |  |  |  | 1 | 1 |  |  |  |  |  |  |  |  |  |  |  |  |  |  |  |  |  | T5 |
| 120 |  |  |  |  |  |  |  |  |  |  |  |  |  |  |  |  |  | 1 |  |  |  |  |  |  |  | T258 |
| 121 | 1 |  | 1 |  |  |  |  |  |  |  |  |  |  |  |  |  |  |  |  |  |  |  |  |  |  | T228 |
| 122 |  |  |  |  |  |  |  |  |  |  |  |  |  |  | 1 |  |  |  |  |  |  |  |  |  |  | T252 |
| 123 |  |  |  |  |  |  |  |  |  |  |  |  |  |  |  |  |  |  |  |  |  |  | 1 |  |  | T305 |
| 124 |  | 1 |  |  |  |  |  |  |  |  |  |  |  |  |  |  |  |  |  |  |  |  |  |  |  | T64 |
| 125 | 1 |  |  |  |  |  |  |  |  |  |  |  |  |  |  |  |  |  |  |  |  |  |  |  |  | T179 |
| 126 |  |  |  |  |  |  |  |  |  |  |  |  |  |  |  |  |  |  |  | 1 |  |  |  |  |  | T333 |
| 127 |  |  | 1 |  |  |  |  |  |  |  |  |  |  |  |  |  |  |  |  |  |  |  |  |  |  | T261 |
| 128 | 1 |  |  |  |  |  |  |  |  |  |  |  |  |  |  |  |  |  |  |  |  |  |  |  |  | T118 |

Table S4. Presence-absence matrix of OTUs (operational taxonomic units) on trees colonised by *Azteca brevis*. Presence-absence matrix for 62 OTUs of carton fungi on 19 trees (Tm: *Tetrathylacium macrophyllum*, Lo: *Lonchocarpus* sp.) at five collection sites, differing by at least 13 mutations. “Seq ID” is the sequence number in the phylogenetic tree (Figure S1) representing one OTU.

| OTU | Bird Trail – Lodge | | | | |  | Río Sardinal | | | |  | Waterfall | |  | Río Sardinal | | | |  | Río Bolsa | |  | Río Gamba |  | Waterfall | Seq ID |
| --- | --- | --- | --- | --- | --- | --- | --- | --- | --- | --- | --- | --- | --- | --- | --- | --- | --- | --- | --- | --- | --- | --- | --- | --- | --- | --- |
|  | Tm1 | Tm2 | Tm3 | Tm4 | Tm5 |  | Tm6 | Tm7 | Tm8 | Tm9 |  | Tm10 | Tm11 |  | Tm12 | Tm13 | Tm14 | Tm15 |  | TmA | TmC |  | TmE |  | Lo |  |
| 1 | 1 | 1 | 1 | 1 |  |  |  | 1 | 1 | 1 |  |  |  |  | 1 | 1 | 1 |  |  |  | 1 |  | 1 |  |  | T66 |
| 2 |  | 1 |  | 1 |  |  |  |  | 1 | 1 |  |  |  |  |  |  |  |  |  |  |  |  |  |  |  | T111 |
| 3 | 1 | 1 | 1 | 1 |  |  |  |  | 1 | 1 |  |  |  |  |  | 1 |  |  |  |  | 1 |  |  |  | 1 | T8 |
| 4 |  |  |  |  |  |  |  |  |  |  |  |  |  |  |  |  |  |  |  |  |  |  |  |  | 1 | T316 |
| 5 |  |  | 1 | 1 | 1 |  |  | 1 | 1 | 1 |  |  | 1 |  | 1 |  |  |  |  |  | 1 |  |  |  |  | T121 |
| 6 |  |  |  |  |  |  |  |  | 1 |  |  |  |  |  | 1 |  |  |  |  |  |  |  |  |  | 1 | T255 |
| 7 |  |  |  |  | 1 |  |  |  |  |  |  |  |  |  |  |  |  |  |  |  |  |  |  |  | 1 | T388 |
| 8 |  |  |  |  |  |  |  |  |  |  |  |  |  |  |  |  |  |  |  |  |  |  |  |  | 1 | T318 |
| 9 |  |  |  |  |  |  |  |  |  |  |  |  |  |  |  |  |  |  |  |  |  |  |  |  | 1 | T314 |
| 10 |  |  | 1 | 1 |  |  |  |  |  |  |  |  |  |  |  | 1 |  |  |  | 1 |  |  |  |  |  | T202 |
| 11 |  |  |  |  |  |  |  |  |  |  |  |  |  |  |  |  |  | 1 |  |  |  |  |  |  |  | T149 |
| 12 |  | 1 |  | 1 |  |  |  |  |  |  |  |  |  |  |  |  |  | 1 |  | 1 | 1 |  |  |  |  | T78 |
| 13 |  |  |  |  |  |  |  |  |  |  |  |  |  |  |  |  |  |  |  |  |  |  |  |  | 1 | T392 |
| 14 |  |  |  |  |  |  |  |  |  |  |  |  |  |  |  |  |  | 1 |  |  |  |  |  |  |  | T140 |
| 15 | 1 | 1 | 1 |  |  |  |  |  | 1 | 1 |  |  |  |  |  |  |  |  |  |  |  |  | 1 |  |  | T358 |
| 16 |  | 1 |  |  |  |  |  |  |  |  |  |  |  |  |  |  |  |  |  |  |  |  |  |  |  | T58 |
| 17 | 1 | 1 |  |  |  |  | 1 |  | 1 |  |  |  |  |  | 1 | 1 |  | 1 |  | 1 | 1 |  |  |  |  | T84 |
| 18 | 1 |  |  |  |  |  |  |  |  |  |  |  |  |  |  |  |  |  |  |  | 1 |  |  |  |  | T50 |
| 19 |  | 1 |  |  |  |  |  |  |  |  |  |  |  |  |  |  |  |  |  |  |  |  |  |  |  | T243 |
| 20 |  | 1 |  |  |  |  |  |  |  |  |  |  |  |  |  |  |  |  |  |  |  |  |  |  |  | T242 |
| 21 |  |  |  |  |  |  |  |  |  |  |  |  | 1 |  |  |  |  |  |  |  |  |  |  |  |  | T367 |
| 22 |  |  |  |  |  |  |  |  |  |  |  |  |  |  | 1 |  |  |  |  |  |  |  |  |  |  | T171 |
| 23 |  |  | 1 |  |  |  |  |  |  |  |  |  |  |  |  |  |  |  |  |  |  |  |  |  |  | T22 |
| 24 |  |  | 1 |  |  |  |  |  |  |  |  |  |  |  |  |  |  |  |  |  |  |  |  |  |  | T239 |
| 25 |  |  |  |  |  |  |  |  |  |  |  |  |  |  |  | 1 | 1 |  |  |  |  |  |  |  |  | T136 |
| 26 |  |  |  |  |  |  |  |  |  |  |  | 1 |  |  |  |  |  |  |  |  |  |  |  |  |  | T366 |
| 27 |  |  |  |  |  |  | 1 |  |  |  |  |  |  |  |  |  |  |  |  |  |  |  |  |  |  | T246 |
| 28 |  |  | 1 |  |  |  |  |  | 1 |  |  |  |  |  |  |  |  |  |  |  |  |  |  |  |  | T210 |
| 29 | 1 |  | 1 |  |  |  |  |  |  |  |  |  |  |  |  |  | 1 |  |  |  |  |  |  |  |  | T223 |
| 30 |  |  |  |  |  |  | 1 |  |  |  |  |  |  |  |  |  | 1 | 1 |  |  |  |  |  |  |  | T221 |
| 31 | 1 |  |  |  |  |  |  |  |  |  |  |  |  |  |  |  |  |  |  |  |  |  |  |  |  | T259 |
| 32 | 1 |  |  |  |  |  |  |  |  |  |  |  |  |  |  |  |  |  |  |  |  |  |  |  |  | T117 |
| 33 |  |  |  |  |  |  |  |  |  |  |  |  |  |  |  |  |  |  |  |  |  |  |  |  | 1 | T394 |
| 34 |  |  |  |  |  |  | 1 |  |  |  |  |  |  |  |  |  |  |  |  |  |  |  |  |  |  | T245 |
| 35 |  |  |  |  |  |  |  |  |  |  |  |  |  |  |  |  | 1 |  |  |  |  |  |  |  |  | T175 |
| 36 |  | 1 |  |  |  |  |  |  |  |  |  |  |  |  |  |  |  |  |  |  |  |  |  |  |  | T192 |
| 37 |  | 1 |  |  |  |  |  |  |  |  |  |  |  |  |  |  |  |  |  |  |  |  |  |  |  | T222 |
| 38 |  |  |  |  |  |  |  |  |  |  |  |  |  |  | 1 |  |  |  |  |  |  |  |  |  |  | T279 |
| 39 | 1 |  |  |  |  |  |  |  |  |  |  |  |  |  |  |  |  |  |  |  |  |  |  |  |  | T428 |
| 40 |  | 1 |  |  |  |  |  |  |  |  |  |  |  |  |  |  |  |  |  |  |  |  |  |  |  | T190 |
| 41 |  | 1 |  |  |  |  |  |  |  |  |  |  |  |  |  |  |  |  |  |  |  |  |  |  |  | T430 |
| 42 |  |  |  |  |  |  |  |  |  |  |  |  |  |  |  |  |  |  |  |  |  |  | 1 |  |  | T357 |
| 43 | 1 | 1 | 1 |  |  |  |  |  |  |  |  |  |  |  |  |  |  | 1 |  |  |  |  |  |  |  | T97 |
| 44 | 1 |  |  |  |  |  |  |  |  |  |  |  |  |  | 1 |  |  | 1 |  |  |  |  | 1 |  |  | T122 |
| 45 |  | 1 |  |  |  |  |  |  |  |  |  |  |  |  |  |  |  |  |  |  |  |  |  |  |  | T61 |
| 46 | 1 |  | 1 | 1 |  |  |  |  |  |  |  |  |  |  |  |  |  | 1 |  |  |  |  |  |  |  | T11 |
| 47 |  |  | 1 |  |  |  | 1 |  |  |  |  |  |  |  |  |  |  |  |  |  |  |  |  |  |  | T7 |
| 48 |  |  | 1 |  |  |  |  |  |  |  |  |  |  |  | 1 |  |  | 1 |  |  |  |  |  |  |  | T9 |
| 49 | 1 |  | 1 |  |  |  | 1 |  |  |  |  |  |  |  |  |  |  |  |  |  |  |  |  |  |  | T182 |
| 50 |  |  | 1 |  |  |  |  |  |  |  |  |  |  |  |  |  |  |  |  |  |  |  |  |  |  | T200 |
| 51 |  | 1 |  | 1 |  |  |  |  |  |  |  |  |  |  |  |  |  |  |  |  |  |  |  |  |  | T98 |
| 52 | 1 | 1 | 1 | 1 |  |  |  |  |  |  |  |  |  |  | 1 |  |  | 1 |  | 1 |  |  | 1 |  |  | T23 |
| 53 | 1 | 1 | 1 | 1 |  |  |  |  |  |  |  |  |  |  | 1 |  |  | 1 |  |  | 1 |  | 1 |  |  | T33 |
| 54 | 1 |  | 1 |  |  |  |  |  |  |  |  |  |  |  |  |  |  | 1 |  |  | 1 |  |  |  |  | T29 |
| 55 |  |  |  |  |  |  |  |  |  |  |  |  |  |  |  |  |  |  |  |  |  |  |  |  | 1 | T362 |
| 56 |  |  | 1 |  |  |  | 1 | 1 |  |  |  |  |  |  |  |  |  |  |  |  |  |  |  |  |  | T10 |
| 57 | 1 |  | 1 |  |  |  | 1 | 1 |  |  |  |  |  |  | 1 |  |  | 1 |  |  |  |  |  |  |  | T5 |
| 58 |  | 1 |  |  |  |  |  |  |  |  |  |  |  |  |  |  |  |  |  |  |  |  | 1 |  |  | T305 |
| 59 | 1 |  |  |  |  |  |  |  |  |  |  |  |  |  |  |  |  |  |  |  |  |  |  |  |  | T179 |
| 60 |  |  |  |  |  |  |  |  |  |  |  |  |  |  |  |  |  |  |  | 1 |  |  |  |  |  | T333 |
| 61 |  |  | 1 |  |  |  |  |  |  |  |  |  |  |  |  |  |  |  |  |  |  |  |  |  |  | T261 |
| 62 | 1 |  |  |  |  |  |  |  |  |  |  |  |  |  |  |  |  |  |  |  |  |  |  |  |  | T118 |

# References

De Hoog GS, Gerrits van den Ende AHG (1998) Molecular diagnostics of clinical strains of filamentous Basidiomycetes. Mycoses 41: 183–189.

Jaklitsch WM, Voglmayr H (2011) *Nectria eustromatica* sp. nov., an exceptional species with a hypocreaceous stroma. Mycologia 103: 209–218.

Mayer VE, Voglmayr H (2009) Mycelial carton galleries of *Azteca brevis* (Formicidae) as a multi-species network. Proc R Soc B 276: 3265–3273.

Schoch CL, Seifert KA, Huhndorf S, Robert V, Spouge JL, Levesque CA, Chen W, Fungal Barcoding Consortium (2012) Nuclear ribosomal internal transcribed spacer (ITS) region as a universal DNA barcode marker for *Fungi*. Proc Natl Acad Sci 109: 6241–6246.

Vilgalys R, Hester M (1990) Rapid genetic identification and mapping of enzymatically amplified ribosomal DNA from several *Cryptococcus* species. J Bacteriol 172: 4238–4246.

Voglmayr H, Rossman AY, Castlebury LA, Jaklitsch WM (2012) Multigene phylogeny and taxonomy of the genus *Melanconiella* (*Diaporthales*). Fungal Divers 57: 1–44.

White TJ, Bruns T, Lee S, Taylor J (1990) Amplification and direct sequencing of fungal ribosomal RNA genes for phylogenetics. In: Innis MA, Gelfand DH, Sninsky JJ, White TJ, editors. PCR Protocols: a guide to methods and applications. San Diego: Academic Press. pp. 315–322.
